# Cigarette brand diversity and price changes during the implementation of plain packaging in the United Kingdom

**DOI:** 10.1111/add.14282

**Published:** 2018-06-25

**Authors:** Magdalena Opazo Breton, John Britton, Yue Huang, Ilze Bogdanovica

**Affiliations:** ^1^ University of Nottingham UK Centre for Tobacco and Alcohol Studies Division of Epidemiology and Public Health Nottingham UK

**Keywords:** Brands, cigarettes, market, plain packaging, price, smoking

## Abstract

**Background and aim:**

Plain packaging of cigarettes appeared in the United Kingdom in July 2016 and was ubiquitous by May 2017. The change coincided with another legislative change, raising the minimum pack size from 10 to 20 cigarettes. Laws imposing plain packaging on cigarette packs remove another promotional route from tobacco companies, but the effect of such laws on brand diversity, pricing and sales volume is unknown. This study aimed to (1) describe and quantify changes in brand diversity, price segmentation and sales volumes and (2) estimate the association between the introduction of plain cigarette packaging and cigarette pricing in the United Kingdom.

**Design:**

We used a natural experiment design to assess the impact of plain packaging legislation on brand diversity and cigarette prices. The data comprised a sample of 76% of sales of cigarettes in the UK between March 2013 and June 2017.

**Setting:**

United Kingdom.

**Measurements:**

Cigarette prices, number of brands and products and volumes of sales.

**Findings:**

During the period analysed, there was a slight decrease in the number of cigarette brands. There was also an initial increase observed in the number of cigarette products, due mainly to an increase in the number of products in packs of fewer than 20 cigarettes sold before July 2016, which was then followed by a rapid decrease in the number of products that coincided with the implementation of the new legislation. Cigarette sales volumes during this period did not deviate from the preceding secular trend, but prices rose substantially. Regression results showed that price per cigarette, regardless of pack size, was 5.0 [95% confidence interval (CI) = 4.8–5.3] pence higher in plain than in fully branded packs. For packs of 20 cigarettes, price increases were greater in the lower price quintiles, ranging from 2.6 (95% CI = 2.4–2.7) GBP in the lowest to 0.3 (95% CI = 0.3–0.4) GBP per pack in the highest quintile.

**Conclusions:**

The implementation of standardized packaging legislation in the United Kingdom, which included minimum pack sizes of 20, was associated with significant increases overall in the price of manufactured cigarettes, but no clear deviation in the ongoing downward trend in total volume of cigarette sales.

## Introduction

Globally, approximately 7 million people die from smoking every year 1. The World Health Organization Framework Convention on Tobacco Control identifies key policies to reduce smoking prevalence, which include the use of tax to reduce the affordability of tobacco, and of standardized or ‘plain’ packaging to reduce product appeal 2, 3. The United Kingdom has a strong record in tobacco control policy 4 and in 2016 became the first country in Europe, and second in the world, to enact legislation mandating plain packaging for all tobacco products. From 20 May 2016, this legislation required all tobacco products branded, manufactured or imported for the UK market to be in packs of a standard green–brown colour, with branding limited to a name and single descriptor in a standard font 5; after a 12‐month transition period which ended on 20 May 2017, this applied to all tobacco products sold in the United Kingdom. The legislation was implemented alongside the 2014 European Union Tobacco Products Directive, which for cigarettes imposed a minimum pack size of 20 and prohibited flavours and misleading product descriptors 6, and in a context of high cigarette prices 7 sustained by annual tax increases of 2% above inflation since 2013 and the introduction of minimum excise tax in May 2017 8.

Tobacco packaging has been used extensively by the tobacco industry to promote brand names, appeal to young people through novelty designs and flavours, distract attention from health warnings and to build and sustain brand equities that act as a ‘silent salesman’ to attract new customers and generate brand loyalty among established smokers 9, 10, 11, 12, 13, 14. Branding is also crucial to the pricing models used by the tobacco industry to maximize profits 15, and in particular the use of higher profit margins on premium products to absorb and hence reduce the effect of tax increases on the affordability of brands in the lower end of the tobacco price spectrum, which tend to be favoured by the most price‐sensitive smokers 16. The new UK plain packaging legislation may therefore not only reduce the appeal of cigarettes, but also, if consumers prove less willing to pay for premium brands in plain packs, result in higher prices (and hence reduced affordability) for the cheapest brands. This study aimed (1) to describe and quantify changes in brand diversity, price segmentation and sales volumes; and (2) estimate the association between the introduction of plain cigarette packaging and cigarette pricing in the United Kingdom.

## Methods

### Design

We used Nielsen Scantrack cigarette sales data from March 2013 to June 2017 to analyse product diversification, volume of cigarette sales and pricing descriptively, and a regression model to estimate the association between plain packaging and cigarette prices.

### Data

Nielsen Scantrack cigarette sales data include monthly volume of sales, value of sales, units sold and average retail price for products scanned from more than 75 000 megastores, superstores, high street stores and convenience stores in the United Kingdom. The total volume of sales by year obtained in Nielsen Scantrack data represents approximately 76% of government figures on annual cigarette consumption in the United Kingdom 17.

### Measures

#### Cigarette products

We defined cigarette products as unique combinations of the following characteristics:

- Brand (for example: Allure, Berkeley or Benson & Hedges)
- Brand variant (for example: slim, king size, silver, gold, menthol, capsule)
- Pack size (the number of cigarettes per pack)
- Multi‐pack size (the number of packs sold together in a multi‐pack)

Thus, for example, one product would be a single pack of 20 Marlboro Gold King Size cigarettes; another might be a multi‐pack of five packs of 19 John Player Special Blue Superkings cigarettes. Product diversification was defined by the number of brands, brand variants and products (combination of brand, brand variants, pack size and multi‐pack size) available each month during the study period.

#### Price

Monthly average retail prices were computed by Nielsen, by dividing the value of sales by the number of units sold using total coverage (all store types) data for each product. Prices of products sold in branded packs were defined as either standard or promotional, the latter applying if the retail price was reduced by 5% or more from the second highest price for the same product observed in the preceding 6 weeks. From July 2016 a separate price category was introduced for products sold in plain packs.

Two price outcomes were defined for the regression analysis: the average retail price per cigarette, which included all available products in all pack sizes, and the average retail price for products sold in 20‐cigarette packs, which were the most common and the only legal pack size available after plain packaging implementation.

#### Volume of sales

Volume of sales referred to the number of cigarette sticks sold each month for each product in the data set. Because Nielsen categorizes products based on their price (standard, promotional and plain pack), volume of sales by price category was computed. ‘Branded’ volume of sales included the number of cigarette sticks sold in both standard and promotional price packs, while ‘total’ refers to the number of cigarette sticks sold in standard, promotional and plain packs.

#### Adapted name

During the period after the European Parliament formally approved the revised Tobacco Products Directive in February 2014, most of the brand variant names that were prohibited by the Directive were either changed to colour descriptors (for example, menthol became green, full flavour became red, and smooth became sky blue or bright blue) or were given a new descriptor (such as real, original, legendary or capsule) to identify original versions of the product 18. Examples of these changes include Players Superkings Menthol, which became Players Superkings Green; Carlton Smooth Blue, which became Carlton Bright Blue; Superkings, which became Superkings Original Black; and Marlboro Ice Blast, which became Marlboro Ice Blast Capsule. Based on this, we created two variables related to changes in brand or brand variant names: an ‘adapted name’ indicator variable with the value ‘1’ for brand variants whose name changed after February 2014 and ‘0’ for those that did not; and a ‘plain pack adapted name’ indicator variable which allocated a value of ‘1’ to products which appeared with an adapted name for the first time in plain pack (which in all cases was after May 2016).

#### Additional covariates

Following Su *et al*. 19 and Barnett *et al*. 20, we included the following additional covariates in our regression model: costs of production (world nominal monthly price of tobacco in US dollars 21 converted to GBP using monthly exchange rates 22), taxes affecting each product specifically (specific tax for cigarettes in GBP per 1000 sticks 23) and market share of the product (based on the assumption that the tobacco industry is not perfectly competitive as proposed elsewhere 19, 20, and computed by dividing the product's volume of sale to total volume of sales in the market using the Nielsen Scantrack Dataset).

#### Subgroup by pack size and price segment

Price segmentation has been identified in the literature as an industry strategy to overshift increases in prices 16, while pack size has been identified as an instrument to increase sales 24 and affect cigarette consumption 25. For the analysis by pack size we categorized products by the number of cigarettes in a pack (10, 11–19 and 20 or more) and the number of packs (single or multi‐pack). For the analysis by price segment we defined price quintiles (quintile 1 comprising the lowest and quintile 5 the highest‐priced cigarettes) from monthly distributions of standard pack prices for each product in fully branded packs (promotional pack prices were excluded), and once established for a fully branded product, applied to the same product in a plain pack. As there were some minor fluctuations in quintiles over time (for example, price fluctuations could result in a product being included in ‘quintile 2’ in one month and in ‘quintile 3’ in the next month), we used the mode of monthly quintiles to assign each product to one quintile. As all plain cigarette packs contained 20 cigarettes, analysis by price quintile was restricted to products that were available in single packs of 20 cigarettes per pack.

### Statistical analysis

#### Descriptive analysis

For the descriptive analysis we used line graphs to compare changes in product diversification, volume of cigarette sales and pricing between fully branded and plain pack products before, during and after plain packaging implementation. Subgroup analysis by pack size and price segment was performed.

#### Regression analysis

As price per cigarette and price per pack for all 20 cigarettes pack exhibited an approximately normal distribution in a histogram, we used linear regression to estimate the association between plain packaging and cigarette price. We used a regression model based on the assumption that as advertising is banned for all tobacco products in the United Kingdom (comprehensive ban on tobacco promotion in 2003, advertising ban at point of sale in 2004, ban on brand sharing and sponsorship in 2005, tobacco vending machine ban in 2011 and, finally, the point of display ban in large vendors in 2012 and in small vendors in 2015 26), the only marketing strategies left for the industry to communicate to consumers 27, 28 and affect prices 29, 30 are related to the product's name or to its package. The regression model estimated price changes associated with the adoption of plain packaging for each product, taking into account changes in brand variant names that took place before plain packaging implementation (‘adapted name’) and during plain packaging implementation (‘plain pack adapted name’).

We first modelled price per cigarette for all products using the key predictor identifying plain packaging products, the two variables related to adaptation of the product name and covariates for pack size, costs of production, taxes and market share. This model allowed us to estimate the mean difference in price between plain pack products and fully branded products, taking into account that fully branded products in a range of pack sizes, and plain pack products exclusively in 20 cigarette‐packs, coexisted in the market during the transition period. We then modelled price per pack using a similar specification but restricting the analysis to packs of 20 cigarettes. Both models were adjusted by year, month and product. Subgroup analysis by price quintile were carried out in the second model. All analysis was performed using Stata version 15 and the confidence level was set to 95%.

## Results

The data set included a total 58 190 valid observations from 1064 products, with an average of 658 fully branded (range = 431–824) and 138 plain pack products (range = 8–226) per month followed‐up for approximately 30 months each (range = 1–52 months).

### Appearance of plain packs on the UK market

**Figure 1:**
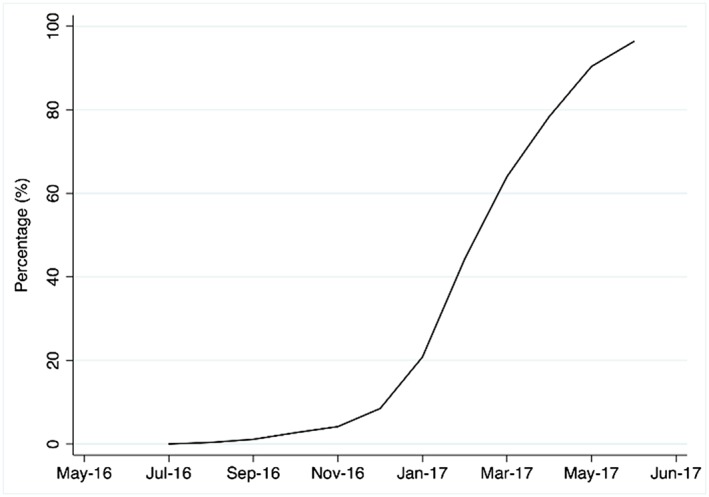
Implementation of plain packaging in the United Kingdom (May 2016–June 2017): monthly plain pack sales volume as a proportion (%) of all sales. [Colour figure can be viewed at http://wileyonlinelibrary.com]

The first cigarette products in plain packs appeared on the market in July 2016 (Fig. 1). The proportion of products in plain packs increased slowly until February 2017, and then rapidly to reach 96% of the total volume of sales in June 2017.

### Number of brands and products

**Figure 2:**
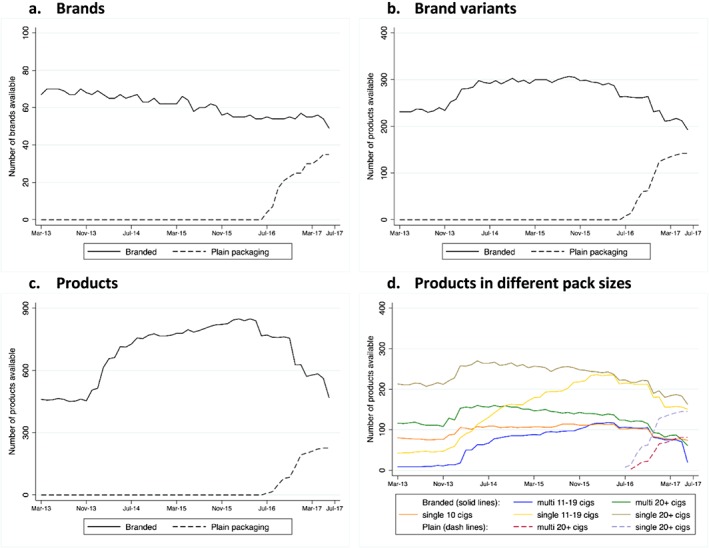
Numbers of brands, brand variants, products and products in different pack sizes, by month and by branded or plain pack, United Kingdom 2013–17.

There was a slow but sustained decline in the total number of brands available on the market between March 2013 and June 2017 (Fig. 2a), but in early 2014 the number of brand variants (Fig. 2b) and products (Fig. 2c) available for sale increased substantially. The increase in product numbers up to July 2016 arose primarily from an increase in the number of single packs containing between 11 and 19 cigarettes, with a smaller increase and more stable trends over time in other pack sizes (Fig. 2d). Single packs of 20 cigarettes or more accounted for the greatest number of products in the market from the beginning of 2014 until the end of the study period.

The number of brands available in fully branded packs decreased during the study period, and by June 2017 the number of brands available in plain packs was still lower than the number of brands available in fully branded packs (Fig. 2a). Similar trends were observed in the number of brand variants (Fig. 2b). The largest difference between plain pack and fully branded products was found for the number of products (combination of brand, brand variant, pack size and multi‐pack size) (Fig. 2c), which was attributable primarily to the disappearance of products in packs of fewer than 20 cigarettes (Fig. 2d). By June 2017 the number of products in fully branded and plain packs of 20 and more cigarettes had reached similar numbers, both for single and multi‐packs.

### Volume of sales and monthly average price per cigarette

**Figure 3:**
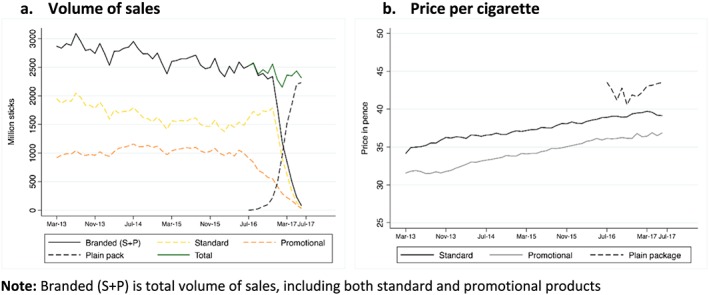
Volume of sales and average price per cigarette, by month, 2013–17. Branded (S + P) is total volume of sales, including both standard and promotional products.

The total number of cigarettes sold in the market decreased continuously from March 2013 (Fig. 3a), and by June 2017 the volume of cigarette sales in plain pack products was consistent with a continuation of that trend. This general trend reflected primarily a decrease in volume of sales of standard priced products, which represent 63% of the total volume of sales, and decreased from 1.9 in March 2013 to 1.5 billion sticks in July 2016. Conversely, promotional price products represented an average of 37% of the total volume of sales and exhibited a relatively constant level at approximately 1 billion sticks until June 2016 (Fig. 3a). The average price per cigarette in fully branded packs increased progressively, from 34 to 39 pence for standard priced products, and from 31 to 37 pence for promotional priced products, between March 2013 and June 2017. The average price of a cigarette in a plain pack was consistently higher than the average price, in the same month, of a cigarette in a fully branded pack and in June 2017 was 43.5 pence (Fig. 3b).

### Subgroup analysis by pack size

**Figure 4:**
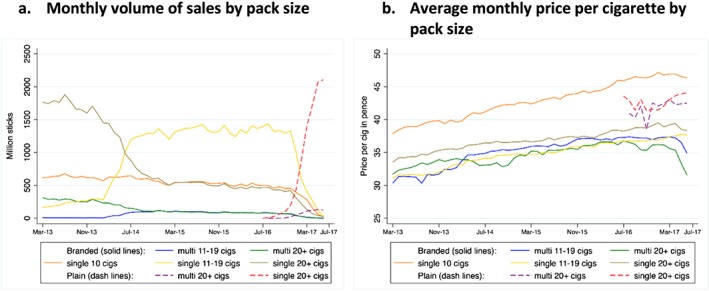
Cigarette sales and average monthly price per cigarette by pack size.

From July 2014 until January 2017, sales volumes were dominated by packs of 11–19 cigarettes (Fig. 4a). Analysis of price per cigarette by pack size suggests that the highest‐priced cigarettes were sold in fully branded single packs of 10 cigarettes, and the lowest in multi‐packs of all sizes, and then in single packs of 11–19 cigarettes (Fig. 4b). The price per cigarette in plain pack products was lower than that of fully branded packs of 10 cigarettes, but higher than for all other fully branded products (Fig. 4b).

### Subgroup analysis by price quintile

An average of 45 products in single packs of 20 cigarettes per pack were followed through time for each quintile. Because plain pack products were introduced gradually during the transition period after May 2016, the number of plain pack products that could be compared to fully branded products increased slowly from July 2016 until the end of the study period. Only approximately half of all fully branded products had a plain pack counterpart by June 2017.

**Figure 5:**
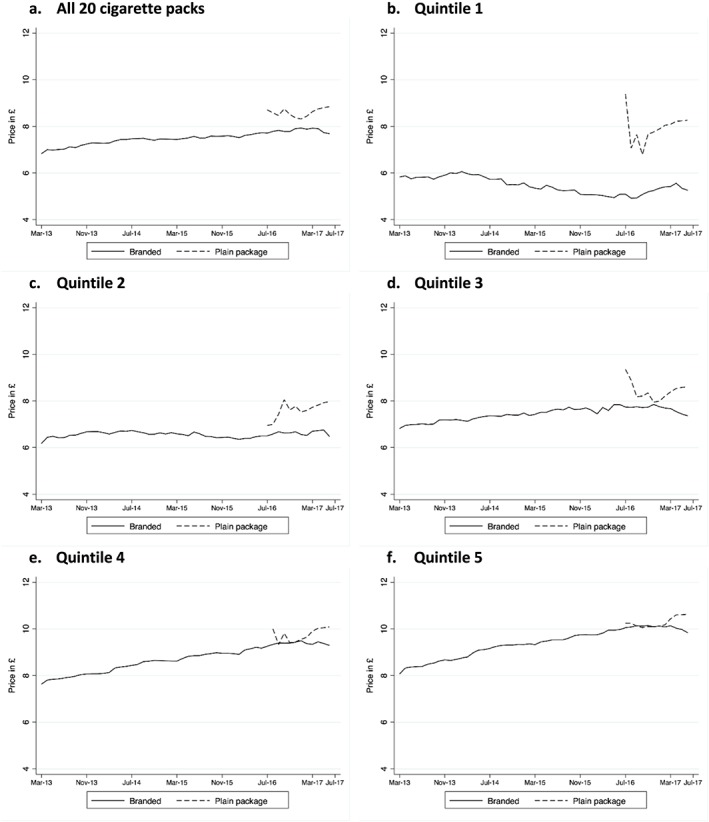
Monthly average price per pack for branded and plain pack cigarettes for all products and by price quintile (1 = lowest, 5 = highest). [Colour figure can be viewed at http://wileyonlinelibrary.com]

The difference in price between products in fully branded and plain packs of 20 cigarettes was largest in the lowest price quintile (an average difference of 2.5 GBP per pack in quintile 1) and decreased gradually with increasing quintile average price to a difference of 0.9 GBP per pack in quintile 5. Lower‐priced cigarettes thus became considerably more expensive with the adoption of plain packs, while the price of the most expensive products remained relatively stable (Fig. 5a–f; see Regression analysis for further details).

**Figure 6:**
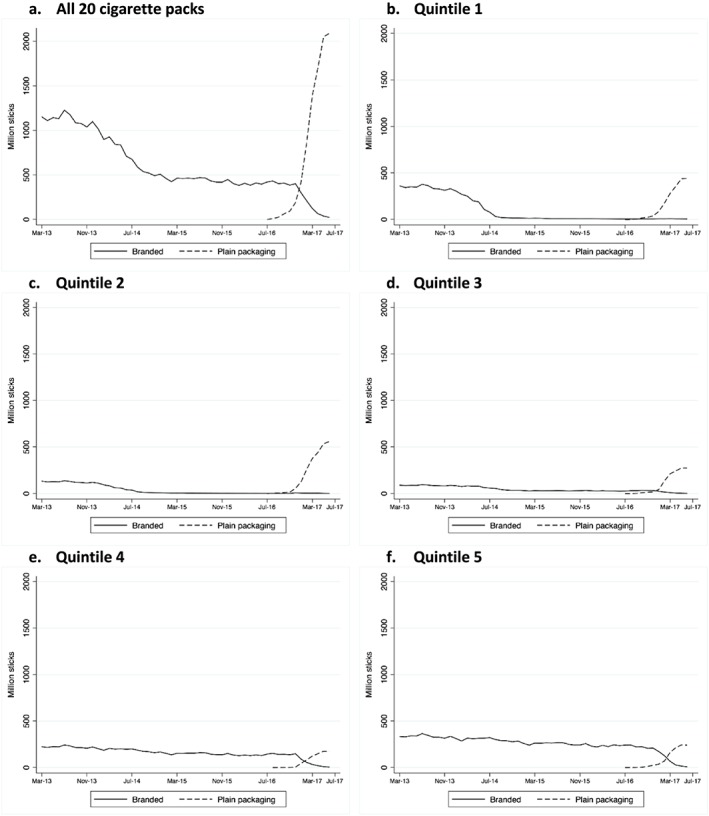
Monthly volume of sales for branded and plain pack cigarettes for all products and by price quintile (1 = lowest, 5 = highest). [Colour figure can be viewed at http://wileyonlinelibrary.com]

Volumes of sales decreased considerably from early 2014 onwards for cigarettes in packs of 20 in the lowest price quintile (quintile 1) from a total of 397 million sticks in February 2014 to 5 million sticks in June 2017, and in quintile 2 from 133 million sticks in February 2014 to 2 million sticks in June 2016 (Fig. 6), although this is an artefact of the near withdrawal of 20‐pack cigarettes (and replacement with packs of 19 or fewer) in these lowest‐priced quintiles during this period. This coincides with the observed increase in volume of sales in cigarettes sold in packs of 11 to 19 cigarettes (Fig. 4a). However, after the withdrawal of all packs of fewer than 20 cigarettes at the end of the transition period, sales volumes in the lowest price quintiles increased again, probably as a result of consumers of smaller packs switching to the cheapest available packs of 20 or more cigarettes. Volumes of sales in the most expensive quintiles showed little change (Fig. 6a–f).

### Regression analysis

Results from our first regression model, involving all pack sizes, suggest that cigarettes in plain packs were on average 5.0 pence [95% confidence interval (CI) = 4.8–5.3) more expensive than standard‐priced fully branded packs (Table 1). There was no statistically significant difference in price for products that adapted their names after February 2014, or for products that appeared with an adapted name in plain packaging. Therefore, in the regression model using all products in the market, plain packaging was associated with a significant increase in price.

**Table 1:** Association between plain packaging and price per cigarette in the United Kingdom (March 2013–June 2017).

	Mean difference^a^	P‐value	95% CI
Plain pack	5.0	< 0.001	4.8 to 5.3
Adapted name	−1.0	0.312	−3.0 to 1.0
Plain pack adapted name	−0.1	0.939	−1.8 to 1.7
Observations			35 869

a Mean difference in price per cigarette in pence obtained using the following covariates: total number of cigarettes in the pack, costs of production, taxes, market share of the product, product ID, year and month. CI = confidence interval.

The regression model using only packs of 20 cigarettes per pack also confirmed that the price per pack was higher for plain packaged compared to fully branded products (Table 2). The indicator variable identifying products that adapted their name after February 2014 showed a negative association with price, meaning that products that adapted their name had a 2.8 GBP (95% CI = 2.9–2.7 GBP) lower price per pack compared to products that did not adapt their name after February 2014. However, the price of those products that appeared in the market in plain packaging with an adapted name after May 2016 was 1.2 GBP higher (95% CI = 1.1–1.4).

**Table 2:** Association between plain packaging and prices per pack for single packs of 20 cigarettes and by price quintile in the United Kingdom: mean differences^a^ (March 2013–June 2017).

	All 20 packs	Quintile 1 (lowest price)	Quintile 2	Quintile 3	Quintile 4	Quintile 5 (highest price)
Plain pack	1.0 (< 0.001) [0.9 to 1.1]	2.6 (< 0.001) [2.4 to 2.7]	1.2 (< 0.001) [1.0 to 1.4]	0.7 (< 0.001) [0.5 to 0.9]	0.4 (< 0.001) [0.3 to 0.5]	0.4 (< 0.001) [0.3 to 0.4]
Adapted name	−2.8 (< 0.001) –2.9 to −2.7	–	−0.7 (< 0.001) –1.0 to −0.4	0.6 (< 0.001) 0.5–0.8	–	0.3 (0.048) 0.0 to 0.7
Plain pack adapted name	1.2 (< 0.001) [1.1 to 1.4]	–	–	–	–	–
Observations	12 372	2532	2409	2162	2389	2449

a Mean difference in price per pack in GBP obtained using the following covariates: costs of production, taxes, market share of the product, product ID, year and month. *P*‐value in parenthesis and 95% confidence intervals in squared brackets.

Finally, the regression by price quintile of products that transitioned from branded to standard packs of 20 cigarettes showed an increase in price per pack associated with the adoption of plain packaging, which was greater in the lower price quintiles and highest in quintile 1 (2.6 GBP; 95% CI = 2.4–2.7).

## Discussion

The United Kingdom is only the second country in the world to introduce plain packaging for cigarettes. This is the first study, to our knowledge, to explore changes in the diversity of cigarette products, volume of cigarette sales and pricing occurring in advance of and during the implementation of this legislation in the United Kingdom, which was implemented alongside pack size and product descriptor restrictions imposed by the European Union Tobacco Products Directive 6. Although the implementation of plain packaging legislation was not associated with any obvious major change in the volume of cigarettes sales, the new legislation was associated with significant increases in cigarette prices, particularly for cigarettes in the lowest quintiles of the price distribution. Our analysis also demonstrates that the implementation of the new legislation was associated with a reduction in the number of products available on the market, reversing a marked increase that occurred in early 2014, and that this was due predominantly to the disappearance of products in packs of fewer than 20 cigarettes. While our data are purely observational and we are unable to attribute causation in the associations we describe, our findings provide an indication of the market and price changes that might be anticipated in other countries adopting similar legislation.

The price increases observed were substantially greater than those attributable to general price inflation in the United Kingdom, which averaged 2.2% (for food, alcohol and tobacco) during the period studied 31 or to the increase in tobacco duty (by 1.16 pence per cigarette) in the March 2017 budget 32. The introduction of a minimum excise tax on 20 May 2017 32 will have caused a differential additional increase in the price of the lowest‐price cigarettes of 35 pence per pack of 20 cigarettes 33, which would mean an increase of 1.75 pence per cigarette. Therefore, the withdrawal of some of the cheapest cigarette packs from the market due to minimum pack size legislation and the increase in price associated to plain packaging have, together, resulted in an overall reduction in the affordability of cigarettes, and particularly for the lowest‐price products.

Our analysis is based on Nielsen Scantrack data collected from a sample of shops and which allow us to identify standard, promotional and plain pack products, but do not specify whether promotional priced products were sold in plain or fully branded packs. However, our descriptive analysis demonstrates clearly that the volume of sales of fully branded and promotional products decreased rapidly towards the end of the study period, while volume of sales of plain pack products increased. This therefore suggests that promotional prices are likely to refer to fully branded packs only.

Our study does not include an analysis of trends in use of hand‐rolling tobacco or illicit tobacco, both of which provide an alternative and much cheaper means of continuing to smoke tobacco for smokers who are sensitive to cigarette price increases. However, the fact that the trend in total cigarette sales volumes remained largely unchanged during the transition to plain packs suggests that there were no major net increases in consumption of hand‐rolling or illicit tobacco products during this period. It therefore appears that despite a substantial price increase, smokers who previously purchased branded packs of low price cigarettes, which were typically sold in packs of fewer than 20 have, predominantly at least, simply migrated to the lowest‐priced cigarettes in packs of 20 at a higher price.

Our findings are broadly in line with data from Australia, suggesting that the introduction of plain packaging led to an increase in cigarette prices 34, whereas the tobacco industry argued that plain packaging would lead to falling prices, downtrading to cheaper products and greater consumption of illicit tobacco 35. However, the sustained consumption of plain pack cigarettes in our study, which occurred in the face of higher prices and particularly at the lower, and hence most price‐sensitive spectrum of the market, contradicts the wide body of price elasticity evidence suggesting that in these circumstances consumption would be expected to fall 36, 37, 38. A possible explanation is, however, that the introduction of plain packaging has proved to be a disincentive to the illicit market, as illicit packs, by virtue of being branded, are now more obviously illegal. Although the estimated size of the illicit market in the United Kingdom increased slightly in 2016/7, figures including the final months of the transition period, when the proportion of plain packs on the market grew most quickly, are not yet available 17.

The aim of the current study was to quantify the immediate changes in cigarette market and pricing during the transition to plain packaging. However, having transitioned to plain packs, minimum pack sizes and restrictions on brand descriptors and flavours over a relatively short period of time, the tobacco market will continue to evolve and adapt to this regulatory environment. Our analysis of prices by quintile was complicated by the fact that the lowest‐priced cigarettes were, before 2016, predominantly in packs of fewer than 20, all of which were withdrawn with the introduction of plain packs. Therefore, the effects of plain packaging and minimum pack size are completely confounded in our data. However, branded packs of 19, 18 or fewer cigarettes often appear to have been priced just below perceptual price points (for example, at a price of £6.99) and it may be that the inability to manipulate pack size has reduced capacity to adopt this practice, and led the tobacco companies to stop competing to undercut these price points. It remains to be seen whether, after the market disruption caused by the introduction of plain packaging, the higher price and relatively restricted product structure that has emerged will persist. Additional work will therefore be required to explore the short‐term impact of the policy on cigarette demand and the longer‐term effects of increases in price and changes in products availability both on the tobacco market and on general population's health. In line with this, it will be important to explore trends in the hand‐rolling tobacco market, and their interactions with trends in manufactured cigarette use, and we are in the process of carrying out these analyses. Regarding short‐term effects on cigarette demand, more research will be needed in order to estimate correctly the effect of the policy on cigarette demand accounting simultaneously for the effects in price observed in our results and the change in tobacco product packaging enforced by the new legislation.

The reasons for the marked increase in product availability in early 2014 are not clear, but this increase followed very quickly the agreement by the European Parliament of draft legislation for the 2014 TPD on 18 December 2013 39. The increase was mainly attributable, however, to products in packs of fewer than 20 cigarettes, which the new TPD prohibited, making it possible that the tobacco industry saw this period as a last opportunity to appeal to young and price‐sensitive smokers by offering a wider range of affordable cigarettes, and hoping that many of these smokers would continue to smoke cigarettes after the TPD was implemented. If so, it appears that their strategy led to switching between various sizes of cigarette packaging but did not succeed in increasing cigarette use in the United Kingdom.

There are at least three potential explanations for the observed increase in prices we observed in relation to plain packaging. First, it is possible that the price increases were a response by the industry to the minimum tax per pack of cigarettes from 20 May 2017 32, using gradually introduced higher prices to cushion the apparent magnitude of the effect of the new minimum tax. Secondly, it is possible that rising prices reflect a commercial strategy of focusing more on premium products or to the loss of opportunity to use pricemarks (which are prohibited under the plain packaging legislation) to highlight price discounts 40. Thirdly, retailers may also have taken the opportunity presented by the prohibition of pricemarks to raise prices and hence their profit.

Our data allow us to conclude, however, from this early experience of plain packaging and TPD policies in the United Kingdom, that the new policies appear to have generated a substantial reduction in the range of cigarette products available for sale and increased cigarette prices, particularly at the lower end of the price distribution. Whether this will translate into a reduction in smoking prevalence or simply to further downtrading into handrolling or illicit tobacco remains to be established.

## Declaration of interests

None.
